# Developing a rare disease registry for inherited metabolic disorders in Pakistan

**DOI:** 10.1186/s13023-026-04510-3

**Published:** 2026-07-24

**Authors:** Lena Jafri, Hafsa Majid, Sibtain Ahmed, Azeema Jamil, Saba Abdul Mateen, Akhtar Shah, Aysha Habib Khan

**Affiliations:** Section of Chemical Pathology, Department of Pathology and Laboratory Medicine, Aga Khan University, Karachi, Pakistan

**Keywords:** Rare, Inherited metabolic disorders, Amino acids, Organic acids, Registry

## Abstract

**Background:**

Inherited metabolic disorders (IMDs) underrecognized and underreported in Pakistan because of fragmented data systems, limited surveillance, and constrained diagnostic resources. We aimed to establish a continuous registry for IMDs to generate structured epidemiological, clinical, and biochemical data in a genetically heterogeneous population with high rates of consanguinity.

**Methods:**

A single-center rare disease registry for IMDs was developed at the Biochemical Genetics Laboratory, Aga Khan University Hospital, Pakistan, and implemented between 2024 and March 2026 using retrospective consolidation of cases from January 2013 onward, followed by prospective real-time data capture. Demographic, clinical, and biochemical data were collected through structured case report forms and linked laboratory records. Registry performance was monitored using predefined indicators, including case volume, number of diagnostic categories, and data quality assessed through three audit cycles, 12 staff training rounds, and source verification procedures.

**Results:**

The registry included 3,880 unique cases from January 2013 to March 2026 and documented 59 distinct IMDs across all four provinces of Pakistan. Parental consanguinity was reported in 66.5% of cases (*n* = 2,584). The most frequently identified disorders were methylmalonic aciduria (*n* = 555), biotinidase deficiency (*n* = 287), and urea cycle disorders (*n* = 218). The final data dictionary comprised 14 categories and 384 common data elements. Quality control measures, including 12 training rounds and periodic audits, improved data accuracy and completeness from 60% to 96% to enable successful registry integration. Piloting data collection tools achieved > 90% completeness in demographic and clinical fields.

**Conclusion:**

This first registry for IMDs in Pakistan provides a structured, quality-assured epidemiological resource for defining the burden and spectrum of these disorders. The registry establishes a foundation for improved diagnosis, service planning, policy development, and future multicenter rare disease research.

**Clinical trial number:**

Not applicable.

## Background

Health-related data are gathered using environmental monitoring, questionnaires, notifications, surveillance and registries [1, 2]. A disease registry is a system that collects standardized data from patients diagnosed with specific diseases, to monitor outcomes, evaluate treatment effectiveness, and ensure safety [3, 4]. Healthcare registries vary in scope and may support research, quality improvement, surveillance, patient safety, utilization analysis, outcome evaluation, longitudinal follow-up, quality-of-life assessment, population health, and regulatory compliance [5, 6].

Rare diseases collectively are a major public health concern, even though each one is individually uncommon. The low prevalence of rare diseases can limit clinicians’ knowledge and expertise in managing these conditions. Registries support research on rare diseases by detailing their natural history, supporting medication research, enhancing clinical trial recruitment, and developing tools to improve care quality [7]. Multiple international registry models exist for rare diseases, including disease-specific registries focusing on individual conditions (e.g., the International Collaborative Gaucher Group, European Cystic Fibrosis Society Patient Registry, and TREAT-NMD network for Duchenne muscular dystrophy and spinal muscular atrophy), consortium or network registries that gather data for research collaboration (e.g., Orphanet, a European network that comprehensive rare disease nomenclature and is a primary index for over 790 registries globally, and the Rare Diseases Clinical Research Network), population surveillance registries (e.g., EUROCAT for congenital anomalies, EURACAN for rare adult solid cancers across 24 European countries, and France’s national registry Banque Nationale de Données Maladies Rares for rare diseases), and reference network registries such as the European Reference Networks [8–16]. These registries differ in design and technological sophistication, often employing electronic data capture and standardized terms like phenotype-based ORPHA codes. Some are centralized, aggregating data across multiple sites, while others are single center but provide valuable real-world data. Rare disease registries globally vary from large multi-country networks to specialized disease and national surveillance systems.

In low- and middle-income countries (LMICs) such as Pakistan, limited diagnostic capacity, low disease awareness, inequitable access to care, and resource constraints impede the diagnosis of rare diseases [17–21]. In the absence of centralized clinical data, disease burden and progression remain poorly characterized, limiting informed policy development and resource allocation. Urban-rural disparities, a weak research ecosystem, and limited engagement with precision medicine further widen these gaps. Given Pakistan’s high consanguinity rate, the burden of rare diseases, especially inherited metabolic disorders (IMDs), is likely substantial but remains largely undefined owing to the absence of a national newborn screening (NBS) program and adequate diagnostic facilities [22]. Pakistan also lacks a national plan for rare diseases. The present registry addresses a key gap addresses a key gap by generating foundational epidemiologic and clinical data on IMDs in an underrepresented population, supporting future inclusion in global collaborative efforts.

## Methods

### Study setting and design

This single-center IMD registry was developed at the Biochemical Genetics Laboratory (BGL), Section of Chemical Pathology, the Aga Khan University Hospital (AKUH), Karachi, Pakistan. The BGL has provided diagnostic services for high-risk patients with IMDs since 2012. Through a nationwide network of more than 300 collection stations, the Clinical Laboratories receives specimens from all provinces of Pakistan, providing broad geographic coverage. The specimens are accompanied by a standardized clinical history form, which served as the paper-based case report form (CRF) for the registry. This study was conducted and reported according to the REporting of studies Conducted using Observational Routinely collected health Data (RECORD) statement, an extension of the STROBE guidelines for registry-based research.

### Governance and ethical oversight

**Fig. 1:**
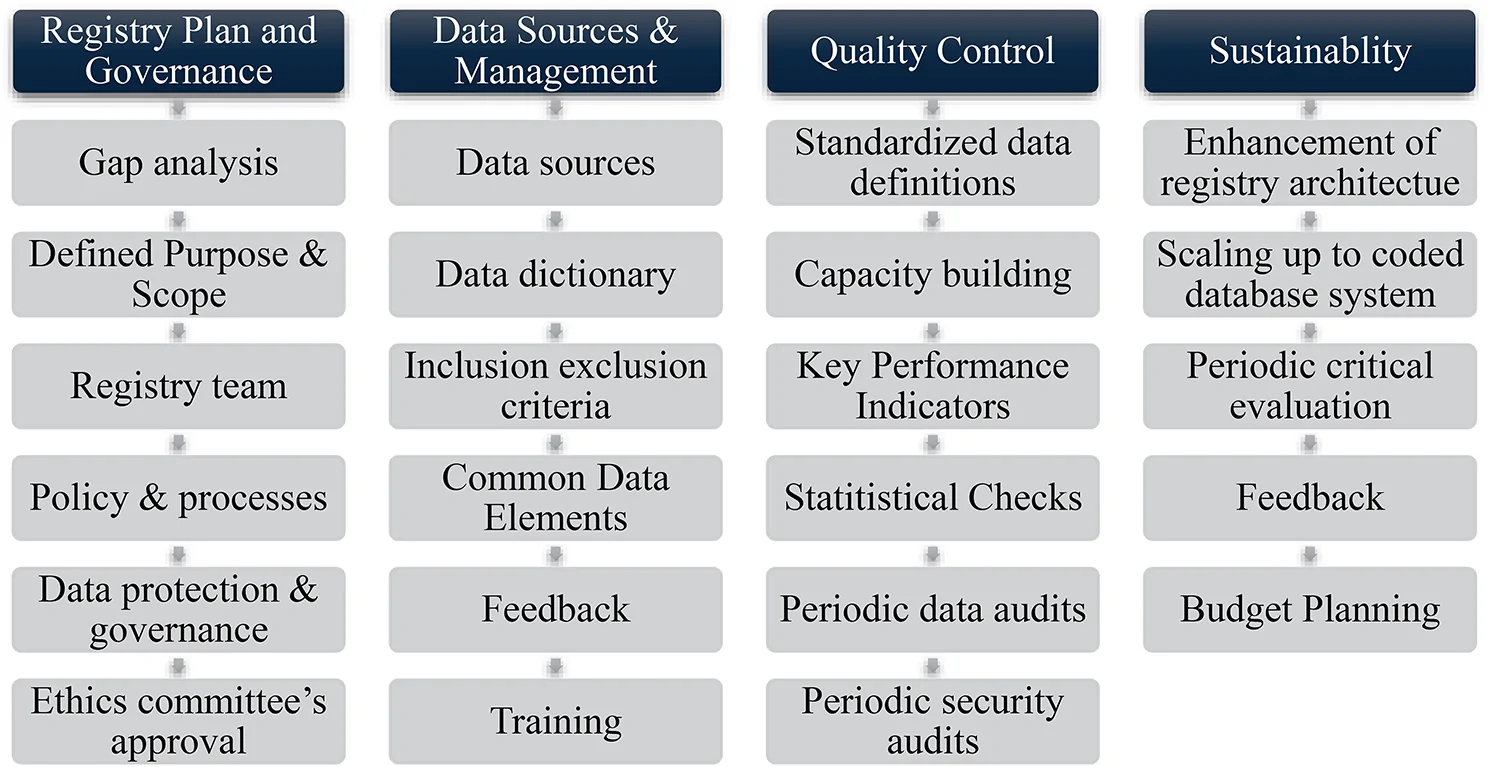
Functional components of rare registry development for inherited metabolic disorders

As illustrated in Fig. 1, registry development followed a structured governance framework. A multidisciplinary team comprising laboratory managers, chemical pathologists, clinical scientists, BGL technologists, and quality assurance representatives was established. A funded data operations position and a dedicated Rare Disease Registry Management Team supported implementation and continuous quality improvement. Oversight was provided by the Section Head of Chemical Pathology, who was responsible for overseeing.

The registry proposal and data collection procedures were approved by the Aga Khan University Ethical Review Committee, which waived informed consent owing to the registry’s non-interventional observational design and the impracticality of obtaining consent for each entry. Data requests were managed according to a standard operating procedure requiring a defined purpose, compliance with institutional policies, and ethical approval where applicable. A digital audit trail ensured accountability, and external data sharing required formal agreements to safeguard patient privacy. Microsoft Excel was selected because of budgetary constraints and its compatibility with existing workflows.

### Eligibility criteria and data sources

The registry aimed to collect clinical and diagnostic data on IMDs, describe the demographic and clinical spectrum, and generate local epidemiological evidence to guide care and planning. The registry included patients with IMDs identified based on biochemically positive screening results, including semi-quantitative urine organic acids on GCMS, quantitative plasma amino acids on HPLC, quantitative urinary succinyl acetone values on GCMS, plasma methylmalonic acid (MMA), and dried blood spot (DBS) acylcarnitine and amino acids on LCMSMS. Inclusion was based on biochemical genetics evidence and not clinical suspicion alone. Repeat samples from the same patient and cases with secondary metabolic abnormalities were excluded. Patient data were captured using CRFs completed for each sample received in the BGL (Table 1). These CRFs served as the primary source of clinical information, while the final IMD diagnosis was confirmed through integration with BGL and laboratory information system records.

**Table 1:** Paper based Case Report Form (standardized clinical history form) with streamlined data entry and high phenotypic detail

Patient Data (History and Profile)	Disease Data (Clinical and Diagnostics)
Case identifier	Signs & symptoms of current illness
Gender	Feeding history
Date of birth	Developmental history
Age at sampling	Significant Past history
Physician’s name	Physical examination
Geographic location	Provisional diagnosis
Significant antenatal history	Treatment if given
Significant birth history	Metabolic Screen: Blood gases & electrolytes, Liver function tests, Glucose, Ammonia & Lactate, Hmocysteine, Urinary biochemistry
Family history	Cultures: CSF biochemistry and culture & Blood culture
Parent consanguinity	Imaging: CT Brain, MRI Brain

### Data elements and dictionary development

All essential common data elements (CDEs) were defined through the development of a comprehensive data dictionary in Microsoft Excel. Standardized formats, controlled vocabularies, and coding systems ensured consistent terminology and precise variable definitions. The registry team finalized the CDEs and data dictionary through repeated meetings to establish consensus on clinical relevance and feasibility. The team integrated data from multiple sources, including CRFs, BGL reports, raw instrument outputs, pathologist interpretations, and newborn screening data where available. The data dictionary specified the purpose, coding values, interpretations, and inclusion or exclusion criteria for each element, thereby guiding both data entry and analysis.

### Data management, coding, and verification

To support interoperability and standardization, disease-specific coding based on standard clinical nomenclature was applied for more than fifty disorders and conditions via controlled data entry fields. Standardized International System of Units, specifically micromoles per liter (µmol/L), were strictly required for marker entries. The quantitative values were further categorized using standardized, semi-quantitative clinical assessment criteria (classified as mild, moderate, or severe) based on predefined biomarker thresholds. These entries were strictly enforced using spreadsheet data validation constraints to minimize input errors. Each case record included a standardized diagnostic interpretation field. Data accuracy was operationally defined as the percentage of error-free records verified against source clinical documentation. To execute this, a formal double-entry audit procedure was conducted, where two independent team members verified 100% of the case records, resolving any data discrepancies by consensus to ensure complete validation. The registry was maintained in a secure, password-protected flat-file structure where each row represented a unique patient observation identified by a pseudonymized registry ID.

### Quality assurance procedures

Data operators received in-house training on registry workflow, data entry, and review procedures. To preserve accuracy during manual transfer from paper records and the laboratory information system into the registry, a double-entry validation process was used. Data were routinely checked for accuracy, consistency, and completeness, and a continuous quality improvement plan with defined key performance indicators (KPI) was applied (Table 2). As data for KPI assessment were obtained from the Rare Registry Database, Laboratory Information System (LIS), case report forms (CRFs), laboratory worksheets, chromatogram reports, and registry access logs. These sources were used to evaluate data quality and governance performance across the registry lifecycle. Each registered IMD case was reviewed against a quality checklist, and case records, laboratory worksheets, and chromatogram reports were examined for alignment with diagnostic standards. Missing fields, absent laboratory identifiers, misclassified diagnoses, and incomplete metabolite profiles were logged for correction. Monitoring activities were conducted at predefined intervals, including monthly, quarterly, and biannual assessments, depending on the indicator being evaluated. Random unannounced audits were also performed for governance-related indicators such as data security and privacy compliance.

**Table 2:** Key performance indicators for rare registry audit framework

Domain	KPI	Operational Definition	Measurement Unit	Data Source	Frequency
Data Quality	Data Completeness	The proportion of required data elements that are filled in correctly in the registry for every record.	%	Rare Registry Database	Random check on 2 cases monthly
	Coverage Completeness	Comparing the eligible cases included in the registry against the total number of cases identified from the source population (LIS)	%	Laboratory information system (LIS)	Quarterly
Data Quality	Data Timeliness	The time lag between laboratory report generation and data entry in registry	Days from report generation to registry entry	Laboratory information system (LIS) and Rare Registry Database	Biannual
Data Quality	Data Accuracy	Proportion of randomly selected patient records and data elements that match the corresponding values in CRF and LIS	%	Laboratory information system (LIS), CRF, laboratory worksheets, and chromatogram reports and Rare Registry Database	Random check on 2 cases monthly
Data Quality	Data Consistency	Degree of uniformity in recording of predefined data elements across different disorders, across various time points and different data entry points for randomly selected diseases	% of discordant entries	Rare Registry Database, CRF, LIS, BGL data	Monthly rotational audit covering all disorders (subset of disorders every month)
Data Quality	Data Validity	The extent to which recorded diagnosis and clinical classification in the registry conforms to predefined case definition	% concordance with expert panel review and diagnostic algorithm	Diagnostic algorithms, clinical records and pathologist panel review	Biannual
Data Governance	Data Security	Protection of registry data from unauthorized access, or loss using system based controls (login access, audit logs).	Number of failed login attempt in Rare Registry Database, Number of data export violations, number of security breaches	Registry access logs, IT system logs	Random unannounced periodic checks by the Section Head/designee (at least biannual) _
Data Governance	Data Privacy	Protection of patient confidentiality through de-identification of data and restricted access to registry following institutional and regulatory requirements for protection of registry data	number of privacy breaches per audit period	Policy, ethics and data protection documentation, registry dataset checks (identifiers removed, access restricted)	Random unannounced periodic checks (at least biannual) _

### Statistical analysis

Statistical analysis was performed in Microsoft Excel for continuous quality monitoring and epidemiological characterization. Descriptive statistics were used to summarize patient demographics and disease-specific clinical and biochemical variables. Epidemiological indicators, including age at diagnosis and point prevalence within the registry, were calculated to identify clinical patterns. Periodic descriptive audits were also conducted to assess completeness and monitor changes in diagnostic trends over time.

## Results

From January 2013 to March 2026, the registry integrated demographic, clinical, and biochemical data for 3,880 cases of IMDs. A summary of the current single-center registry characteristics, including primary methodologies and case ascertainment, is provided according to RECORD guidelines. A comprehensive summary of the registry’s data sources and variable definitions is presented in Table 3.

**Table 3:** Rare registry’s organizational structure in accordance with RECORD guidelines

Record Item	Single-Centre Registry Result; *n* = 3880	Clinical Significance
Data Source	Biochemical Genetics Laboratory at Aga Khan University, Pakistan	Represents a specialized, high-risk screened population.
Study Period	January 2013-March 2026	Rapid accrual reflecting high regional burden.
Case Definition	Presumptive IMD via Metabolic Profiling	Based on pathognomonic metabolite clusters; dual verified by experienced chemical pathologists
Primary Methodology	GC-MS (Urine organic acids and succinyl acetone), Biochrom or HPLC (Plasma and urine amino acids), LCMSMS (Plasma Methylmalonic acid, DBS amino acids and acylcarnitine)	Analysed in single biochemical genetics lab by trained experienced allied health professionals, Uniform methodology across all diagnoses, validated against pattern recognition and age-matched reference intervals
Catchment Area	National (Pakistan)	Data reflects population specific consanguinity and founder effects.
Case Ascertainment	Symptomatic High-Risk Screening	> 80 **%** of cases identified during metabolic crisis ensuring clinical relevance

The dataset was standardized in an Excel-based database that captured key laboratory parameters. This included 283 variables for urine organic acids, both qualitative and quantitative; 79 variables for plasma amino acids derived from urine and plasma concentrations and categorized by degree of elevation or reduction with interpretive diagnostic outputs; additional components for succinyl acetone and plasma MMA, and 20 variables for DBS amino acids and acylcarnitine. This dataset covered cases across all provinces of Pakistan, providing a broad cross-sectional view of IMDs.

Three sequential audit cycles were conducted to assess registry performance and data quality. Preliminary audits identified several gaps, including incomplete clinical documentation, variability in data entry, and failure to record some diagnostic organic acid peaks. Among aminoacidopathies, tyrosinemia type I was initially unrecorded, and phenylalaninemia cases often lacked complete demographic and biochemical information.

Corrective and preventive measures were then implemented. Missing entries were completed, misclassified cases were reassigned, and standardized protocols for chromatogram interpretation and data entry were reinforced. Refresher training was provided for BGL staff, and a structured feedback process was established in which chemical pathologists offered regular guidance and oversight to support accurate interpretation. Inconsistencies in data entry were further reduced through revision and strengthening of the data dictionary.

The data dictionary was updated and validated against standardized definitions and criteria, reducing the likelihood of future errors. Source records were also double-checked against entered data to strengthen accuracy. The final data dictionary included 14 categories and 384 CDEs and undocumented data items were left blank.

The data collection tools were piloted on three BGL staff members and achieved more than 90% completeness in demographic and clinical fields. Across 12 rounds of training and periodic audits, data accuracy and completeness improved from 60% to 96%. Once quality benchmarks were met, historical BGL data were entered, followed by real-time data capture to keep the registry current. A key project outcome was the successful transition of the registry to a real-time data capture system.

The registry documented 59 distinct IMDs across 3,880 cases, with ages ranging from 0 to 28,470 days. A significant proportion of the parents of the affected individuals, 66.5% (*n* = 2,584), displayed a history of consanguinity. With over 100 documented cases in each condition or disease, the most frequent IMDs were methylmalonic aciduria (14.3%,*n* = 555); biotinidase deficiency (7.4%, *n* = 287); urea cycle disorders (5.6%, *n* = 218); hyperphenylalaninemia (4.9%, *n* = 190); tyrosinemia (4.66%, *n* = 181), maple syrup urine disease (4.61%, *n* = 179); Non-ketotic hyperglycinemia (3.89%, *n* = 151), dicarboxylic aciduria (3.87%, *n* = 150); propionic aciduria (3.6%, *n* = 140); and glutaric aciduria (3.5%, *n* = 137), CBS deficiency (3.2%, *n* = 125), 5 oxoprolinase deficiency (2.5%, *n* = 100) and glutaric aciduria Type 1 (2.5%, *n* = 100). Less frequent IMDs (with < 100 count) were documented in the registry but are excluded from this report. Detailed clinical profiles and analysis of these lower-frequency, ultra-rare cohorts fall outside the scope of the current paper and are reserved for a separate, dedicated publication.

## Discussion

This registry addresses the diagnostic challenges faced by patients with IMDs in Pakistan, where high consanguinity, limited newborn screening, and restricted access to biochemical testing contribute to delayed diagnosis. In the absence of a national NBS program, the registry provides structured local data on disease patterns, case characteristics, and diagnostic gaps in a resource-constrained setting [22, 23].

Rare disease registries include disease-specific, consortium, population-surveillance, and reference-network models [8–16]. In comparison with these models, the current registry represents a single-center, laboratory-linked clinical registry designed to address an evidence gap in a resource-constrained setting. Unlike multinational collaborative platforms, its primary strength lies in the structured capture of clinically rich, locally generated data at the point of diagnosis.

This single-institution registry reflects the strengths of registry initiatives in other emerging healthcare settings. These include established single-center referral hub models such as the Queen Rania Al Abdullah Hospital for Children Metabolic Database in Jordan, as well as more recently developed national and collaborative multi-center networks such as the Iran Metabolic Registry and the first national rare disease registry in Libya [24–26]. Although differing in scale and governance structures, these initiatives share a common objective of systematically capturing clinical and diagnostic data for rare diseases in environments where epidemiological information is often limited. The present registry demonstrates that meaningful rare disease surveillance can be initiated at the institutional level, even in the absence of a national registry framework. Our registry is particularly significant for addressing rare autosomal recessive disorders in a high-consanguinity population and provides critical data often overlooked in international datasets. Similar to other pioneering programs in the region, it leverages existing clinical and laboratory resources to establish a sustainable platform for data collection, patient characterization, and research.

A key novelty of this registry lies in its data integration architecture. Unlike conventional single-source tracking databases, this framework represents the first multi-platform model in our region to integrate raw laboratory information system files, phenotypic characteristics, and ontology-based classifications within a single non-proprietary pipeline. By linking diagnostic laboratory results with standardized clinical and phenotypic information, the registry enables more comprehensive characterization of IMDs and facilitates consistent data capture across diverse patient presentations. The use of a non-proprietary architecture reduces implementation costs, minimizes dependence on specialized software, and allows flexible adaptation to local institutional requirements. This pragmatic approach provides an affordable and reproducible framework for decentralized adoption by healthcare and academic institutions in resource-constrained settings, supporting future expansion, multicenter collaboration, and longitudinal data collection.

Beyond its technical design, the registry also has relevance within Pakistan’s evolving rare disease advocacy landscape. Rare disease advocacy is gradually growing, involving professional societies such as the Society for Novel and Rare Diseases (SNARE), PAK-IMD-Net of the Pakistan Society of Chemical Pathology (PSCP), and the Pakistan Society of Medical Genetics (PSMG), as well as student-led initiatives and some parent support groups [27–30]. These organizations aim to improve diagnosis, research, and policy for many rare diseases. Their efforts include advocating for government support that led to the waiving of custom duties on life-saving therapies and implementing advanced screening technologies for early rare disorder detection. Initiatives such as the e-portal ‘Ek-Sath’ (meaning “together” in a local language of Pakistan) provide disease-related information to families [31]. The current registry represents a further step within these emerging efforts to address rare disorders in Pakistan.

Several challenges were encountered during implementation, including limitations in infrastructure, staffing, expertise in rare disease registry development and funding. Awareness of the role and potential value of disease registries was initially limited among some stakeholders, necessitating advocacy efforts and ongoing engagement to promote institutional support. To address these challenges, targeted workshops, training sessions, and multidisciplinary discussions were conducted to strengthen knowledge, build capacity, and foster stakeholder buy-in. The registry was consequently adapted to local realities, leveraging existing laboratory data systems, preexisting CRFs, and readily available digital tools. Although these resource constraints limited the complexity and functionality of the platform, the resulting pragmatic design enhanced feasibility, scalability, and long-term sustainability(7). Embedding the registry within established academic and clinical workflows further strengthened its operational integration and supports its continued use for education, research, and patient care. The experience gained through local implementation of this registry can inform the development of governance structures, data standards, and collaborative partnerships required for broader registry expansion. While global networks such as Orphanet and the European Reference Networks rely on structured EHR extraction and patient-led surveys, registries in resource-limited areas need customized frameworks [11, 14]. Our approach aligns with the global shift toward FAIR data principles by establishing standardized ontologies, while addressing regional infrastructure challenges through low-cost extraction methods, as seen in localized implementations in academic medical centers [32]. While the current rare disease registry serves as a foundational data-capture platform optimized for the Pakistani context, future iterations must systematically align with FAIR (Findable, Accessible, Interoperable, and Reusable) data principles. The FAIR principles enhance data utility and interoperability. Adoption of these standards is critical for transforming the registry from a local database into a machine-readable resource capable of supporting international natural history studies [33]. Achieving global interoperability will require integration of standardized terminologies and ontologies, including LOINC for biomarkers, ORPHANET or ORDO for disease classification, and the Human Phenotype Ontology for phenotypic characterization [34]. Implementing a “FAIR at source” approach would improve the registry’s value not only for local clinical audits but also for AI-driven analysis and multicenter, cross-border rare disease collaborations.

Several limitations should be acknowledged. The absence of molecular and second-line confirmatory testing reduces diagnostic certainty in some cases. As a single-center initiative, the registry may not fully reflect the national picture, particularly in underserved and rural areas. Its customized, non-standardized classification system also limits interoperability, although this enabled rapid initial implementation. The registry currently lacks longitudinal follow-up data, but future phases will introduce consent procedures and outreach to support outcome tracking.

Data from paper records, laboratory information systems, and mass spectrometry are currently consolidated into a spreadsheet framework, which serves as a baseline data pool for future migration into international standard formats. While comprehensive cross-platform harmonization remains a long-term aspiration, immediate success relies heavily on institutional readiness and behavioral change. Implementing a structured action plan that addresses organizational culture ensures sustainable digital transition and system adoption [32]. Consequently, prioritizing cross-departmental governance and early artificial intelligence integration allows the registry to optimize data workflows, secure patient data trust, and gradually refine clinical utility without overwhelming institutional capacity.

Planned next steps include integrating registry with routine laboratory practice and continued support for data entry, expansion from IMDs to rare metabolic disorders, transition to REDCap, improved data security, electronic health record integration, staff training, policy advocacy, and development of annual reports and patient-friendly infographics. At the same time, this model shares common constraints reported across rare disease registries, including funding instability, restricted access to advanced confirmatory diagnostics, and limited interoperability. For this registry to achieve broader research and policy value, the next priority is harmonization with international standards for data elements, terminology, and exchange.

## Conclusion

This registry provides Pakistan’s first structured real-world IMD dataset and highlights disease patterns, diagnostic challenges, and care gaps. It establishes a foundation for improved diagnosis, service planning, and future collaborative research. The findings suggest that the overarching success of such digital health platforms depends less on immediate, resource-intensive technological overhauls and more on fostering institutional readiness and managing cultural adaptation. Long-term success will depend on continued stakeholder engagement, digital infrastructure investment, and policy support. Such institutional registries can serve as important foundational building blocks for future regional or national rare disease networks by generating standardized datasets, fostering stakeholder engagement, and demonstrating the feasibility of registry-based surveillance in resource-constrained settings.

## Data Availability

No additional data are available for sharing beyond what has been presented in the tables within this manuscript.
